# Recruitment and retention of participants from socioeconomically deprived communities: lessons from the Awareness and Beliefs About Cancer (ABACus3) Randomised Controlled Trial

**DOI:** 10.1186/s12874-020-01149-x

**Published:** 2020-11-04

**Authors:** Vasiliki Kolovou, Yvonne Moriarty, Stephanie Gilbert, Harriet Quinn-Scoggins, Julia Townson, Louise Padgett, Sioned Owen, Peter Buckle, Adrian Edwards, Julie Hepburn, Mandy Lau, Maura Matthews, Caroline Mitchell, Richard Neal, Rebecca Playle, Mike Robling, Stephanie Smits, Rob Trubey, Fiona Wood, Kate Brain

**Affiliations:** 1 Centre for Trials Research Cardiff Metropolitan University, Cardiff, Wales CF14 4YS UK; 2 Division of Population Medicine, Cardiff University, Cardiff, UK; 3 Department of Health Sciences, University of York, York, UK; 4 Lead Lay Research Partner Wales Cancer Research Centre, Cardiff, Wales UK; 5 TENOVUS Cancer Care Gofal Canser, Cardiff, Wales UK; 6 Academic Unit of Primary Medical Care, University of Sheffield, Sheffield, UK; 7 Leeds Institute for Health Sciences, University of Leeds, Leeds, UK

**Keywords:** Recruitment, Retention, Socioeconomic deprivation, Community settings, Randomised control trial, Health intervention

## Abstract

**Background:**

Recruitment of research participants poses challenges in socioeconomically deprived areas. The Awareness and Beliefs About Cancer (ABACus) phase 3 Randomised Control Trial recruited adult participants from socioeconomically deprived areas using a combined healthcare/community engagement model. We report the strategies used to successfully recruit and retain our trial participant sample.

**Methods:**

Community and healthcare settings in areas of high socioeconomic deprivation were identified by lay advisors who recruited participants opportunistically or by appointment. Follow-up was done by telephone or post at 2-weeks and 6-months after recruitment, and all participants were offered financial incentives. Qualitative interviews were conducted with lay advisors regarding their experience and reflections.

**Results:**

The lay advisors identified and contacted 107 potential recruitment venues across South and West Yorkshire and South East Wales of which 41.1% (*n* = 42) were opened for recruitment. A total of 234 participants were recruited, with 91% (*n* = 212) retention at 2-weeks and 85% (*n* = 199) at 6-months. Community settings yielded 75% (*n* = 176) of participants. Participants had a mean age of 61.3 years and 63.3% (*n* = 148) were female, with 66% (*n* = 154) resident in the most deprived geographical areas. Lay advisors described recruitment as intensive, although engaging participants was easier in community settings.

**Conclusions:**

The ABACus3 trial achieved recruitment and high retention with a population that is often “hard to reach” or entirely missed in health research. Strategies were specifically tailored to engage the venues and adult residents of highly deprived areas. Future studies recruiting adults living in the most deprived areas might benefit from community recruitment and from collaborating with local gatekeepers who are key to engagement.

*This study adheres to CONSORT guidelines.*

**Trial registration:**

Retrospectively registered with ISRCTN (http://www.isrctn.com/ISRCTN16872545) on 12.01.2018.

**Supplementary information:**

**Supplementary information** accompanies this paper at 10.1186/s12874-020-01149-x.

## Background

The recruitment and retention of adults living in socioeconomically deprived areas, a historically “hard-to-reach” group, can be challenging in health research [1]. Randomised Controlled Trials (RCTs) are frequently used to test the implementation of health interventions in real-world settings but participant samples rarely reflect realistic socioeconomic deprivation patterns [2]. This could lead to interventions failing to reach certain populations when scaled-up for the general public and could potentially widen inequalities in health outcomes.

Health inequalities for cancer are observed in socioeconomically deprived communities where residents tend to have later-stage diagnosis and poorer survival rates for common cancers [3–5]. This is partly a result of prolonged time to symptom presentation due to barriers such as lower cancer symptom awareness and negative beliefs about cancer [6–8]. Therefore, cancer awareness interventions that are inclusive of socioeconomically deprived populations could encourage earlier symptom presentation, ultimately reducing inequalities in cancer survival outcomes [9]. Traditional recruitment strategies using primary and secondary care practitioners and settings may miss the populations who do not regularly engage with these services [9, 10]. Barriers that prevent some populations participating in these settings include deteriorating health, lack of transport, difficulty accessing services and poor understanding of written recruitment materials [11]. Community settings, in contrast to healthcare settings, and the use of tailored approaches for engagement have been effective in reaching and including “hard-to-reach” populations in health research [9, 12–14].

The ABACus3 Randomised Controlled Trial (RCT) tested a cancer awareness intervention tailored and delivered to adults living in deprived communities [15]. In the current paper, we describe a series of tailored recruitment and retention strategies used in the ABACus3 project [16] to engage this population in both healthcare and community settings. We report the recruitment and retention outcomes from the trial and the insights from the lay advisors who undertook recruitment and delivered the ABACus3 intervention to participants. The aim of this article is to present an example of successful recruitment and retention in a trial targeting a “hard-to-reach” population.

## Methods

### Study design

The ABACus3 trial design is described in detail elsewhere [16]. Briefly, the ABACus3 RCT tested a facilitated cancer awareness intervention (the Health Check) in areas of high socioeconomic deprivation [17]. Participants were randomised upon recruitment (baseline) to either the intervention or control (standard care) group on a 1:1 basis. All participants completed a series of questionnaires at baseline which were repeated at 2-weeks and 6-month follow-up points. The recruitment window for the study opened in December 2017 and closed in January 2019.

The study incorporated process evaluation methods and semi-structured face-to-face interviews were conducted with the three lay advisors who randomised the particpants and delivered the intervention (two based in South Yorkshire and one based in South East Wales) before they started and after they finished the recruitment and intervention delivery. Interviews were conducted by fellow ABACus3 researchers (HQS, YM and SG) with the aid of interview topic guides revised throughout the trial. The qualitative results from this trial, including the interviews with the lay advisors, the interviews with study participants and the intervention delivery audio recordings, are presented in a separate process evaluation paper [16].

### Target areas and socioeconomic deprivation

Recruitment took place in two broad geographical areas: South and West Yorkshire (Sheffield, Wakefield, Barnsley, Doncaster, Rotherham) and South East Wales (Merthyr Tydfil and Newport). All neighbourhoods of high socioeconomic deprivation (10% most deprived or 10–20% most deprived) within these areas were identified using standardised indices, namely the 2015 English Index of Multiple Deprivation for South and West Yorkshire (IMD; [18]) and the 2014 Welsh Index of Multiple Deprivation for South East Wales (WIMD; [19]). Indices data were displayed at neighbourhood or Lower-layer Super Output Area level, which roughly equates to postcodes. The process of identifying eligible neighbourhoods allowed targeted searches of local stakeholders, and the postcodes of prospective recruitment venues were checked using the respective IMD/WIMD tools. We sought to recruit from a range of healthcare and community settings in all identified neighbourhoods. Types of settings included GP surgeries, community pharmacies, libraries, social clubs, sheltered housing, homeless service center, community centres and churches.

### Engaging local stakeholders

The ABACus3 lay advisors were trained to deliver the intervention at baseline and facilitated recruitment in the target areas of South and West Yorkshire and South East Wales. They were responsible for communication with key stakeholders, identification of eligible venues, liaising with local gatekeepers, organising recruitment days, enrolling and randomising participants and delivering the intervention. Due to previous ABACus phases recruiting exclusively from South East Wales and in keeping with the funder’s strategic focus on the Yorkshire population, emphasis was placed on recruiting participants from South and West Yorkshire. A database was kept by the lay advisors of all requests to community and healthcare venues, detailing contact attempts made by the trial team and the postcode of the venue. Eligible healthcare and community settings were contacted individually by lay advisors or were sent an Expression of Interest form (for healthcare settings) by local stakeholders supporting the trial. The lay advisors liaised with local stakeholders in healthcare settings: Pharmaceutical Committees (Yorkshire), Clinical Commissioning Groups (Yorkshire) and Welsh University Health Boards (South Wales). The lay advisors also approached local councils governing the most deprived communities intending to start a snowball effect in community settings. Individual healthcare and community venues were contacted via e-mails and phone calls by the lay advisors to introduce and invite participation in the trial as a recruitment venue.

### Opening venues for recruitment

Where willingness to take part was indicated, the lay advisors would visit and meet local gatekeepers at the venues to check venue eligibility– chiefly, a private room to conduct the intervention and a reliable internet connection (needed for the online health check). The lay advisors recorded their reflections of each visit to a recruitment venue using a standardised proforma. These records included details of the day of the week, time of arrival and departure, the type of recruitment session arranged (opportunistic, pre-booked appointments or both), the total number of people recruited at the venue and an estimate of the average footfall at the venue while the advisor was present. There was no provision in the research budget for payments to centres for use of facilities in community or healthcare settings, and in many cases, community settings waived the fee to allow the research to take place. In healthcare settings in South and West Yorkshire, payment was provided by some Clinical Commissioning Groups as part of their remit to support research in primary care; however, the trial team was not involved in any payment arrangements.

Visits before recruitment were used to establish rapport with venue managers and other key staff and encourage a collaborative approach to recruiting local participants. Venue managers and key staff advised on the days when there would be maximum footfall, described the demographic profile of their visitors (e.g. age and language). Those who had capacity to support recruitment further booked provisional appointments with potential participants ahead of the advisors visiting for a recruitment day.

### Discovering and reviewing new venues

The lay advisors searched and opened new healthcare and community settings regularly throughout the recruitment window in order to secure further recruitment days and avoid recruitment stagnation. Following recruitment, the suitability of the opened venue was assessed based on the eligibility of the visitors attending the venue. After eligible visitors were recruited, the English Index of Multiple Deprivation (IMD) or Welsh Index of Multiple Deprivation (WIMD) were used as appropriate to check whether the participant’s postcode was within the target socioeconomic deprivation bracket.

### Sample and inclusion criteria

To adequately power the study, we aimed to recruit 246 participants which would have allowed for an attrition rate of 30% (i.e. 172 participants retained at final follow-up). Adults were considered eligible for inclusion if they were aged 40 or over, and excluded if they were a non-English speaker, unable to give written informed consent or had participated in a previous phase of ABACus.

### Recruitment materials

All patient-facing study materials (participant information sheets, consent forms, questionnaires) were written with consideration to the national average literacy levels and reviewed by the trial’s Patient and Public Involvement group prior to recruitment, to ensure suitability for the target population [16].

### Recruitment of participants

Table 1 summarises the strategies employed to recruit and retain our participant sample in preparation for recruitment and after the participants were recruited.

**Table 1:** Participant recruitment and retention strategies prior to recruitment day, at baseline, 2-weeks and 6-months

Recruitment and retention strategy	Before recruitment	Baseline	2 Weeks	6 Months
Recruitment materials written in accordance with average national literacy levels and reviewed by the trial’s Patient and Public Involvement group.	✓			
Contacted and opened a range of healthcare (GP surgeries, pharmacies) and community (local community groups, community events, one-to-one community sessions) settings for recruitment.	✓			
Venue visitors were offered time slots for participating in future recruitment day(s).	✓			
Participants were asked about their preferred time of week (weekend/weekday), time of day (morning, afternoon, evening) and method of contact (phone call, text message, e-mail, post) for their follow-ups.		✓	✓	✓
Participants were encouraged to include a phone call as preferred method of contact.		✓		
Participants were informed that the trial team (based in Wales) would be calling from a number starting with the 029- telephone code.		✓		
Participants were offered a High Street shopping voucher after completing their questionnaire.		✓		✓
Participants were given an approximate date for their follow-ups.		✓	✓	✓
Emphasis placed on the lay advisors’ affiliation with Cardiff University.	✓	✓	✓	✓

Venue visitors could be recruited through pre-booked appointments or opportunistic recruitment. Pre-booked appointments with interested individuals were made by venue staff or the lay advisors. Opportunistic recruitment was led on site by lay advisors who approached individuals in a community or healthcare setting on set recruitment days agreed with the venue manager or other staff. Interested individuals were assessed for eligibility by the lay advisor and were given the ABACus3 participant information booklet. Once eligibility was confirmed, participants were asked to provide written informed consent before advancing to recruitment, baseline questionnaires and randomisation.

### Follow-up procedures

Once participants were recruited and had completed the baseline questionnaires, a series of strategies was employed to maximise participant retention at 2-weeks and 6-month follow-ups. In addition to the £10 High Street shopping voucher for completing baseline questionnaires, participants were offered a further £5 voucher for completing the final questionnaires at the 6-month follow-up. The lay advisors recorded the participant’s preferred time and method for contact and provided the participant with an approximate date for their next follow-ups. The lay advisor’s affiliation with Cardiff University (as summarised in Table 1) was emphasised to prepare the participants for receiving their follow-up calls.

### Data analysis

**Fig. 1:**
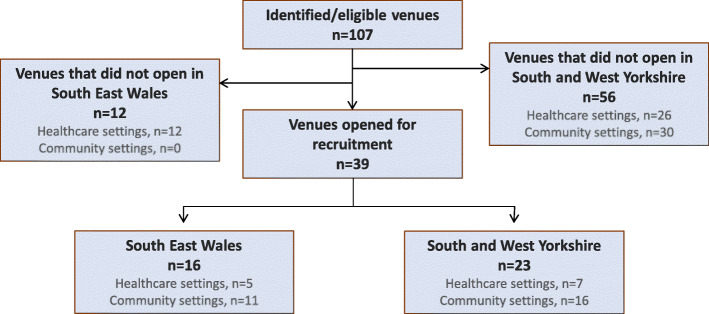
CONSORT diagram illustrating the recruitment settings for ABACus3

**Fig. 2:**
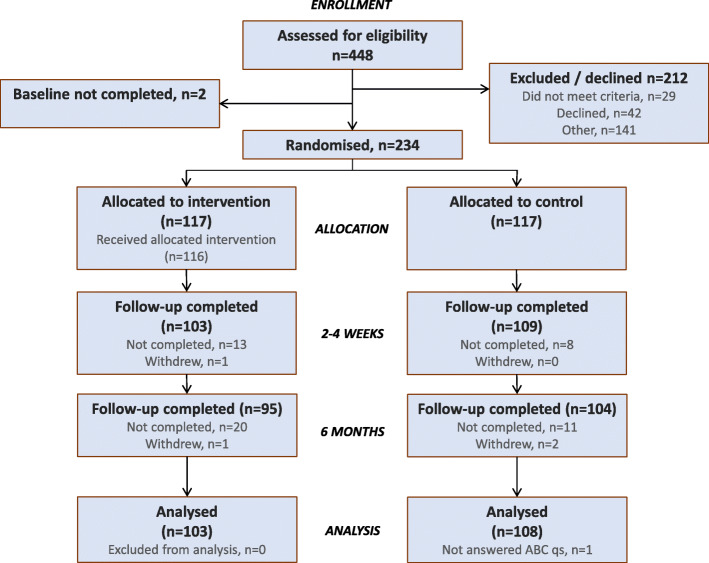
CONSORT diagram of the participant recruitment and retention for ABACus3

Trial recruitment and retention figures were recorded centrally in a study management database, and were presented in accordance with the CONSORT guidelines (Figs. 1 and 2). Descriptive statistics were used to summarise and present data relating to venue and participant recruitment, and participant characteristics. Quantitative data analyses were conducted in Microsoft Excel and SPSS version 25 [20]. Interviews were transcribed verbatim and anonymised for analysis. We used thematic analysis [21] and 100% of lay advisor interviews were double coded by HQS and SG with the aid of the NVivo 11 data management software [22]. The qualitative data will be reported in detail separately, as per the trial protocol [16].

## Results

### Recruitment venues

We identified 107 potential recruitment venues as eligible, of which 79 were in South and West Yorkshire and 28 were in South East Wales, reflecting the study design of requiring two thirds of participants from South and West Yorkshire and one third from South East Wales (Fig. 2). Of the 107 venues, 39 (36%) venues accepted the invitation and opened for recruitment (Fig. 1 and Supplementary Table 1). Appropriate effort was put into reaching both healthcare and community venues, as 41.7% of venue invitations in Yorkshire and 60.7% of invitations in Wales were addressed to healthcare settings (Supplementary Table 1). Opened recruitment venues comprised of 12 (31%) venues in healthcare settings and 27 (69%) venues in community settings (Fig. 1).

### Recruitment days

We conducted 62 recruitment days, of which 44 (71%) days were in community settings and 18 (29%) days were in healthcare settings. Pre-booked appointments were used on 25 (40.3%) days, opportunistic recruitment was used on 30 (48.4%) days, and a combined approach was used on 7 (11.3%) days.

Overall, 448 individuals were assessed for eligibility, with 234 randomised. Of the 214 participants not recruited to the study, 29 did not meet the inclusion criteria and 42 declined to participate (Fig. 2). Common reasons given by members of the public for declining to participate included not being interested in the study (*n* = 42) or not having enough time (*n* = 62).

### Participant sample

Recruitment and retention of trial participants is shown in Fig. 2. There were high retention rates of recruited participants at both 2-weeks (90.5%) and 6-months (85.0%) follow-up points, which meant that the study was adequately powered. Detailed results will be reported separately, as per trial protocol [16].

Table 2 illustrates the participant-reported demographic characteristics of the baseline sample. Almost two thirds of randomised participants were female, half of them had finished school before the age of 16, almost half were retired and almost all were from a white ethnic background. The mean age of the sample was 61.3 years.

**Table 2:** Characteristics of participants, by area and recruitment venue type

Variable		South & West Yorkshire		South East Wales		Combined Areas
		Healthcare settings	Community settings	Healthcare settings	Community settings	Total
		n (%)	n (%)	n (%)	n (%)	n (%)
**Gender**	Male	13 (44.8)	46 (37.4)	8 (27.6)	19 (35.8)	86 (36.7)
	Female	16 (55.2)	77 (62.6)	21 (72.4)	34 (64.2)	148 (63.3)
**Age (years)**	Mean (SD)	64.5 (10.74)	61.6 (11.98)	61.1 (10.19)	58.9 (11.91)	61.3 (11.6)
**Education**	Finished school at/before age 16	14 (48.3)	64 (52.0)	17 (58.6)	24 (45.3)	119 (50.8)
	Completed GCSEs, O-Levels or equivalent	9 (31.0)	21 (17.1)	6 (20.8)	11 (20.8)	47 (20.1)
	Completed A-levels or equivalent	0 (0.0)	9 (7.3)	1 (3.4)	4 (7.5)	14 (6.0)
	Completed further education but not degree	5 (17.3)	22 (17.9)	1 (3.4)	8 (15.1)	36 (15.4)
	Completed Bachelor’s degree/Master’s/PhD	1 (3.4)	7 (5.7)	4 (13.8)	6 (11.3)	18 (7.7)
**Employ-men**t	Employed full-time	3 (10.3)	10 (8.1)	4 (13.8)	9 (17.0)	26 (11.1)
	Employed part-time	1 (3.4)	12 (9.8)	3 (10.3)	5 (9.4)	21 (9.0)
	Full-time homemaker	0 (0.0)	3 (2.4)	0 (0.0)	0 (0.0)	3 (1.3)
	Retired	22 (75.9)	52 (42.3)	13 (44.8)	21 (39.6)	108 (46.1)
	Un- employed	0 (0.0)	22 (17.9)	4 (13.8)	11 (20.8)	37 (15.8)
	Self-employed	0 (0.0)	6 (4.9)	0 (0)	0 (0)	6 (2.6)
	Disabled or too ill to work	3 (10.3)	18 (14.6)	5 (17.2)	6 (11.3)	32 (13.7)
	Prefer not to say	0 (0.0)	0 (0.0)	0 (0.0)	1 (1.9)	1 (0.4)
**Ethnicity**	White	28 (96.6)	122 (99.2)	28 (96.6)	51 (96.2)	229 (97.9)
	White and Black Caribbean	0 (0.0)	0 (0.0)	0 (0.0)	1 (1.9)	1 (0.4)
	White and Black African	0 (0.0)	0 (0.0)	1 (3.4)	0 (0.0)	1 (0.4)
	Pakistani	1 (3.4)	0 (0.0)	0 (0.0)	0 (0.0)	1 (0.4)
	Caribbean	0 (0.0)	0 (0.0)	0 (0.0)	1 (1.9)	1 (0.4)
	Other ethnic group	0 (0.0)	1 (0.8)	0 (0.0)	0 (0.0)	1 (0.4)

Table 3 shows a breakdown of participant deprivation scores by geographic area and type of recruitment venue. Participants were predominantly resident in areas of high socioeconomic deprivation. Of all 234 participants, 154 (66%) had a home postcode that fell within the 10% or 20% most socioeconomically deprived geographical areas in England or Wales according to their respective national deprivation indices (Table 3).

**Table 3:** Summary of individual-level deprivation scores, by area and recruitment venue type

IMD	South & West Yorkshire		WIMD	South East Wales	
	Healthcare n (%)	Community n (%)		Healthcare n (%)	Community n (%)
10% most deprived	16 (55.2)	68 (55.3)	0–10% most deprived	15 (51.7)	16 (30.2)
20% most deprived	2 (6.9)	19 (15.4)	10–20% most deprived	3 (10.3)	15 (28.3)
30% most deprived	2 (6.9)	9 (7.3)	20–30% most deprived	4 (13.8)	9 (17.0)
40% most deprived	3 (10.3)	9 (7.3)	30–50% most deprived	2 (6.9)	7 (13.2)
50% most deprived	0 (0.0)	2 (1.6)	50% least deprived	5 (17.2)	6 (11.3)
50% least deprived	2 (6.9)	7 (5.7)	Total of Recruited Participants	29 (100.0)	53 (100.0)
40% least deprived	0 (0.0)	3 (2.4)			
30% least deprived	2 (6.9)	5 (4.1)			
20% least deprived	1 (3.4)	1 (0.8)			
10% least deprived	1 (3.4)	0 (0.0)			
Total of Recruited Participants	29 (100.0)	123 (100.0)			

### Lay advisor interviews

The lay advisors described that identifying eligible venues, finding the appropriate contact details, arranging meetings with interested venue staff and following up venue staff who had not responded required continuous effort.

“*… the different venues, the different people, it would be really hard to find my way around it, and be difficult to book venues and all that, it would take a while …*”.

*“..sometimes I would just, not necessarily give up, but I would look at other GP surgeries or other community groups that hopefully would be a bit more engaged really, because I knew then if they were quite proactive and quite engaged and wanting to know more about the research the chances are that they would be quite welcoming and try and help with recruitment and stuff like that as well*”.

They considered that engaging venues with the research study and reassuring venues of minimal additional burden were also important to successfully opening venues to recruitment. Lay advisors described the lack of provision within the study budget to pay for venue hire as a barrier to recruiting venues. Some community venues were reluctant to provide a fee waiver for using a private room on recruitment days. Overall, however, the lay advisors expressed that the venue staff considered the trial easy to facilitate.

*“yeah [asking for a fee waiver for room hire] was really the only barrier because up until the point where it came to talking about funding and things like that they were really engaged because it’s cancer research, it’s going to be hard to turn down supporting that and we weren’t really asking a lot of the staff there, we were very low maintenance I think, it was mainly just sometimes the room hire was sometimes a bit of an issue”.*

Interviews with the the lay advisors suggested that finding the direct contact details of the GP manager or responsible staff or having a named contact in healthcare settings was particularly important, rather than cold calling the GP practice receptionist.

*“… yeah, because if you ring up a GP receptionist and start talking to them about research they’re going to want to get you off the phone as quick as possible because they’re got patients ringing, god knows what, so having a name or a direct number does speed things up a little bit, which we got through the research network so yeah it was really useful..”*

They also reflected on engaging the participants on site. The lay advisors expressed the view that community recruitment was easier because of: higher visitor footfall, the visitors having free time on site and being more willing to hear about the trial, and “older visitors” enjoyed talking with the lay advisors.

## Discussion

In this randomised controlled trial of a targeted cancer awareness intervention, we report the strategies used to engage our trial participant sample. The ABACus3 intervention was tailored to increase cancer awareness among adults living in socioeconomically deprived areas in the UK and the trial design was similarly developed to reach and retain this population. Our results show that community-based recruitment, liaising with local gatekeepers and using a personalised flexible follow-up approach were successful strategies for engaging the target population.

Approaches that are designed to encourage participation of socioeconomically deprived populations in research studies are crucial for ensuring that healthcare interventions are inclusive of such groups and have the potential to tackle health inequalities when implemented. The ABACus3 lay advisors forged collaborations with local gatekeepers in community settings and healthcare settings to engage potential participants. This activity required an additional time and resource investment, often because of the fee waiver acting as a barrier, but community recruitment ultimately yielded the majority of trial participants. Recruitment was strengthened by building collaborative relationships with and working alongside venue staff on site, and enabled access to individuals who may not otherwise have engaged with healthcare services. Through their existing relationships with community members, local community gatekeepers were able to promote the benefits of trial participation to individuals who may have been reluctant to take part had they been approached directly by the lay advisors. Prospective participants were also offered a financial incentive which may have contributed to their willingness to participate. The lay advisors also mentioned the value of the participant’s contribution to research by participating and offered a personalised approach for their follow-ups. Follow-ups were arranged to suit the participants availability and preferred method of contact. These strategies may had allowed for added trust and reciprocity between the participants and the researcher lay advisors.

Community-based intervention delivery has been used in other cancer awareness studies and has shown the potential of community members working in partnership with trial staff, operating as local ‘cancer champions’ [15, 23]. Healthcare settings were also included in our recruitment strategy to reach individuals who may not regularly attend community venues due to physical disability, mental health barriers, social exclusion or lack of confidence in attending social venues. Healthcare settings were challenging to approach and open as recruitment venues, possibly due the hierarchy of communication between GP practice staff that needs to be negotiated in order to access the relevant staff member. Engaging healthcare settings to allow the trial access to their patients and facilities was time-consuming due to the complex delegation of responsibilities among staff in GP surgeries. The use of community settings as recruitment centres, coupled with the addition of healthcare venues, lowered recruitment bias in relation to individuals who are not regular visitors to one type of setting but visit the other.

It is important to address the limitations of our recruitment approach. More than half of ABACus3 participants were resident in the 10 to 20% most deprived areas, which was the target population. However, our sample may not be representative of smaller populations within the target population such as members of the BAME community. Analysis of baseline demographic data showed that participants were primarily female and the majority of participants were not in paid employment. Due to limited trial resources, recruitment was restricted to week days and working hours which could have hindered access (particularly in community venues) to working adults. Community venue staff facilitating recruitment through pre-booked appointments may have inadvertently biased recruitment towards visitors who they considered more likely to be interested. Moreover, we did not actively seek to recruit from all neighbourhoods meeting the deprivation criteria, although the search for eligible venues was not restricted. Another study limitation was the lack of translated materials and language support which contributed to limited recruitment of BAME populations, and/or non-English speakers, therefore excluding these populations from the trial. Further research is indicated to establish best practice strategies for involving diverse demographic groups that were not adequately represented in our trial including men, BAME communities and adults from socioeconomically deprived areas in part- or full-time employment.

## Conclusions

Community engagement strategies can improve access to healthcare interventions for adults living in deprived areas. In ABACus3, we used a series of tailored recruitment strategies to recruit and retain a cohort of adults from areas of high socioeconomic deprivation into our health check intervention trial. Recruitment from both healthcare and community venues and the use of flexible, personalised contact strategies were successful in engaging the target population. Although intensive, improving research access for adults living in deprived areas is of paramount importance for health-related research as well as cancer awareness interventions aiming to reduce inequalities in health outcomes.

## Supplementary information

**Additional file 1: Supplementary Table 1:** Summary of invited and opened recruitment venues.

## Data Availability

The data that support the findings of this study are available from the Centre for Trials Research (CTR) in Cardiff University but restrictions apply to the availability of these data, which were used under license for the current study, and so are not publicly available. Data are however available from the authors upon reasonable request and with permission the Centre for Trials Research (CTR) in Cardiff University.

## Abbreviations

ABACus3: Awareness and Beliefs About Cancer phase 3
RCT: Randomised Controlled Trial
BAME: Black, Asian and And Minority Ethnic
