# Supplementary material for: Recruitment and retention of participants from socioeconomically deprived communities: lessons from the Awareness and Beliefs About Cancer (ABACus3) Randomised Controlled Trial
Source: BMC Med Res Methodol. 2020 Nov 4;20:272. doi: 10.1186/s12874-020-01149-x (PMC7641826; doi:10.1186/s12874-020-01149-x)
Supplement: Supplementary file 1 — Additional file 1: Supplementary Table 1: Summary of invited and opened recruitment venues. [file 12874_2020_1149_MOESM1_ESM.docx]

Supplementary Table 1: Summary of invited and opened recruitment venues.

| **Recruitment Venues** | | **Invited**  **n (%)** | **Opened**  **n (% of invited)** |
| --- | --- | --- | --- |
| South and West Yorkshire | Healthcare | 33 (41.7) | 7 (21.2) |
|  | Community | 46 (58.3) | 16 (34.8) |
|  | Total | 79 | 23 (29.1) |
| South East Wales | Healthcare | 17 (60.7) | 5 (29.4) |
|  | Community | 11 (39.3) | 11 (100.0) |
|  | Total | 28 | 16 (57.1) |
| Combined areas | Healthcare | 50 (46.7) | 12 (24) |
|  | Community | 57 (53.3) | 27 (47.4) |
|  | Total | 107 | 39 (36.4) |
